# Visfatin promotes multiple myeloma cell proliferation and inhibits apoptosis by inducing IL-6 production via NF-κB pathways

**DOI:** 10.1007/s12672-025-02682-1

**Published:** 2025-05-20

**Authors:** Wenting Tie, Tao Ma, Jia Liu, Zhigang Yi, Hao Xiong, Jun Bai, Yanhong Li, Lijuan Li, Liansheng Zhang

**Affiliations:** 1 Department of Hematology, The Second Hospital and Clinical Medical School, Lanzhou University, Lanzhou, China; 2 Department of Endocrinology, The Second Hospital and Clinical Medical School, Lanzhou University, Lanzhou, China; 3 Department of Hematology, The Affiliated Hospital of Southwest Medical University, Luzhou, China

**Keywords:** Multiple myeloma, Visfatin, Apoptosis, Interleukin-6, Nuclear factor kappa-B

## Abstract

**Background:**

Multiple myeloma (MM) is the second most prevalent hematological malignancy that results in the proliferation of malignant plasma cells and the overproduction of monoclonal immunoglobulin. Visfatin plays an important role in the regulation of apoptosis, oxidative stress, and inflammation; however, to this date, the role of visfatin in multiple myeloma is unclear.

**Objective:**

To explore the role of visfatin in multiple myeloma and find new targets for MM treatment.

**Methods:**

In this study, expression of visfatin in bone marrow was detected by ELISA. The diagnostic value of visfatin was determined by receiver operating characteristic (ROC) curve analysis. After the quality control by performing western blot to confirm the knockdown of visfatin in two MM cell lines, the phenotype (proliferation and apoptosis) of visfatin in MM was determined by carrying out in vitro experiments, including CCK8, flow cytometry, and western blot. Several cytokines were determined by real-time PCR, followed by in vivo experiments and immunohistochemical assays. IκB, NF-κbp65, and phosphorylation were determined by western blot.

**Results:**

We found that visfatin level increased in the bone marrow of MM patients compared to controls. ROC curve analysis result showed that bone marrow visfatin was able to distinguish MM patients from controls. In vitro and in vivo, visfatin promotes MM cell proliferation. The production of IL-6 was attenuated by visfatin knockdown. Furthermore, we showed that visfatin could activate IL-6 production via the NF-κB signaling pathway.

**Conclusions:**

In MM, visfatin promotes tumor progression by upregulating IL-6 production, which may be a novel therapeutic target for the treatment of MM patients.

## Introduction

Multiple myeloma (MM) is characterized by an excessive accumulation of monoclonal plasma cells in the bone marrow, which disrupts the normal production of blood cells [1]. As a hematologic malignancy, it originates in the immune system's plasma cells and can lead to various complications, including bone damage and anemia. This type of cancer accounts for over 1% of all neoplastic disorders, highlighting its significance in the field of oncology [2]. In the past decades, many effective anti-myeloma treatments have been developed, including proteasome inhibitors (PIs, such as Bortezomib/BTZ), immunomodulatory drugs (IMiDs) to antibody-based, histone deacetylase inhibitors, monoclonal antibodies (mAbs), bispecific antibodies, and chimeric antigen receptor (CAR)-based targeting immunotherapies [3–5].While new treatment classes have significantly enhanced the quality of life for patients with MM, it is important to continue research the etiology of MM, as the disease is still considered incurable. This ongoing effort can pave the way for more effective therapies in the future. The precise etiology of multiple myeloma (MM) is currently not fully understood. However, it is widely acknowledged that a variety of factors, including environmental influences, genetic predispositions, and antigenic stimulation, may play important roles in its development and progression [6]. Further research into these areas is essential for enhancing our understanding and improving interventions for this condition. While age, family history, and monoclonal gammopathy of unknown significance are widely recognized as key risk factors, emerging research suggests that obesity may also contribute to an increased risk of multiple myeloma [1]. This insight emphasizes the potential benefits of addressing weight management as part of a broader strategy for health and disease prevention.

Visfatin is regarded as pre-B cell colony-stimulating factor and nicotinamide phosphoribosyltransferase. Visfatin originally found in visceral fat, is closely related to obesity [7]. It is now clear that visfatin is not only produced by adipocytes but also detectable in immune cells [8], brain cells [9], and cancer cells [10, 11]. It regulates tumor processes such as apoptosis, proliferation, migration, angiogenesis, and invasion [12, 13]. As to MM, visfatin is upregulated in myeloma cells [14]. IL-6, a cytokine with broad function in inflammation and immunity, is recognized as a survival factor for malignant plasma cells involved in MM [15]. The expression of IL-6 in blood and bone marrow in MM patients was significantly greater than that in healthy controls. It has a key role among the cytokines increased in MM patients. IL-6 is delivered by stromal cells and myeloma cells [16]. It acts directly via the JAK/STAT3 signaling pathway to induce the expression of anti-apoptotic protein in MM [17]. IL-6 also confers drug resistance to MM [18]. These studies provided the rationale to evaluate the effects of anti-IL-6 mAb on MM cells.

In the present study, we aimed to explore the role of visfatin in MM. We first examine the expression and diagnostic value of visfatin in MM patients, further verify the role of visfatin in MM through in vivo and in vitro experiments, and explore its possible signal pathways, so as to provide new ideas for the diagnosis and treatment of MM.

## Materials and methods

### Patient samples

Patients’ bone marrow samples were collected from thirty-one newly diagnosed myeloma patients. The diagnosis and efficacy of MM were assessed in accordance with NCCN guidelines [19]. As the control, seventeen cases of iron deficiency anemia excluding tumors were enrolled. The characteristics of the participants were indicated in Table 1. The use of bone marrow specimens and clinical data was approved by the Ethics Committee of the Second Hospital of Lanzhou University (Approval No. 2020 A-106), and written informed consent was obtained from all participants (informed consent was obtained from parents/guardians of minors). All procedures involving human participants performed in this study followed the institutional and/or national research committee’s ethical standards and the 1964 Helsinki Declaration and its later amendments or comparable ethical standards.

**Table 1:** Characteristics of the participants

	Controls	MM patients	*P*
Sex(M/F)	10/7	18/13	0.333
Age(years), median(IQR)	58 (17)	55 (16)	0.715
BMI(kg/m^2^), median(IQR)	22.46 (5.87)	23.18 (5.63)	0.996
IL-2	0.590 (1.490)	0.200 (0.6825)	0.246
IL-6	2.350 (3.882)	7.220 (6.260)	0.001
IL-8	18.53 (44.23)	48.33 (52.88)	0.010
IL-10	1.485 (1.373)	3.090 (5.125)	0.002
IL-17	4.850 (7.88)	2.270 (3.915)	0.216

### Cell culture

Human multiple myeloma AMO-1, RPMI-8226, U266 cells were purchased from the Procell Life Science and Technology Co., LTD (Wuhan, China), grown in RPMI-1640 medium (Tianjin Hao Yang Hua Ke Biotechnology Co., Ltd., China, HY1640) supplemented with 10% fetal bovine serum (FBS, ABW, Germany, AB-FBS-0060S). All cells were cultured in a 37℃ incubator with 5% CO_2_. All cell lines were authenticated by short tandem repeat analysis within 3 years before the study and were regularly checked for mycoplasma contamination[20].

### Cell transfection and clone selection

Lentiviral-mediated pGMLV-short hairpin RNA was from lanzbiotch.cn (Lanzhou, China). Empty vectors were used as the negative control (shNC). MM cells in the logarithmic growth phase were transfected by adding 20 μL 1 × 10^8^ TU/ml lentivirus, culturing in RPMI-1640 medium with 10% FBS in a six-well dish with 2 × 10^5^ cells per well. Lentiviral vectors were transfected into MM cells without the use of pro-transfection reagents. Infected MM cells were screened using puromycin(2 μg/ml) for 6 days. Adjust the cell concentration to 10 cells/ml, and seed 100 μl of cell suspension into each well of a 96-well plate. Then mark single-cell wells, and place the 96-well plate in a 37 ℃, 5% CO_2_ incubator. Select the well where 100% of the cells are fluorescent and have been screened using puromycin. The cell transfection efficiencies were evaluated by western blot [21]. Sequence of Lentiviral-mediated pGMLV-sh-visfatin is shRNA-I: 5ʹ-TACATTCTTGAGAAGTATGA-3ʹ, shRNA-II: 5ʹ-CCTGGCCACCGACTCCTACA-3ʹ, shRNA-III: 5ʹ-GGCTTCCTGGATTTTCTCTT-3ʹ.

### ELISA

Bone marrow samples (3 ml) were collected from the MM patients and controls. The blood samples were centrifuged at 3000 rpm for 20 min, the visfatin of serum was measured by ELISA (Jonln, China) in accordance with the manufacturer’s recommendations. The absorption level was measured with a microplate Reader at 450 nm.

### Cell proliferation assay

Cell counting kit (CCK)−8 (Biosharp, China) was used to assess cell proliferation. Cells (8 × 10^3^) were seeded in 96-well plates and treated with various concentrations of visfatin: 0 ng/ml control, 50 ng/ml, 100 ng/ml, 200 ng/ml. Infected MM cells were seeded into 96-well plates in a final volume of 100 μl of complete culture medium and incubated concentrations for 24 h, 48 h, and 72 h. 10ul of CCK8 solution was added 24 h, 48 h, and 72 h later. To evaluate the proliferation of shNAMPT and shNC cells, cells (8 × 10^3^/well) were inoculated into 96-well plates, we examined the OD values of MM cells at 2.5 h, 24 h, 48 h, and 72 h. After incubation at 37 ℃ for 3 h, the absorbance was measured using an automatic microplate reader (Biotek, USA) at a wavelength of 450 nm (OD 450) at room temperature [22].

### Analysis of cell apoptosis

The cells were treated with or without 100 ng/ml visfatin for 24 h, both the cells were collected by centrifuge and washed with PBS, then stained with Annexin V-FITC (Multi Sciences, China, AP105) for 15 min, 7 AAD (Multi Sciences, China, AP105) for 5 min in the dark at the room temperature, apoptotic cells were detected immediately by flow cytometry. For apoptosis detection, the infected cells were harvested and stained using an Annexin V-APC/7 AAD apoptosis detection kit (Multi Sciences, China, AP105) following the manufacturer’s protocol. FACS analysis was performed by flow cytometry using a flow cytometer (BECKMAN CytoFLEX, USA).

### RNA extraction and quantitative real-time PCR (qRT-PCR)

Totol RNAs were extracted by use of TRIzol reagent (Invitrogen, USA) according to the instructions. The cDNA was synthesized with the Servicebio™ SweScript All-in-one First-Strand cDNA Synthesis SuperMix for qPCR (one-step gDNA Remover) (Serxicebio, China, G3337). IL-6, IL-17, IL-32, and TGF-β mRNA expression in shNC and sh-visfatin were quantified by real-time PCR using SYBR GREEN PCR Master Mix (Servicebio, China, G3321) on real-time PCR system (Thermo Fisher 7500, USA). The primers sequences as follows: IL-6 (Forward primer, 5ʹ-CCTGAACCTTCCAAAGATGGC-3ʹ and reverse primer, 5ʹ-TTCACCAGGCAAGTCTCCTCA-3ʹ); IL-8 (Forward primer, 5ʹ-TCCAAACCTTTCCACCCCAA-3ʹ and reverse primer, 5ʹ-ACTTCTCCACAACCCTCTGC-3ʹ); IL-17 (Forward primer, 5ʹ-TCCCACGAAATCCAGGATGC-3ʹ and reverse primer, 5ʹ-GGATGTTCAGGTTGACCATCAC-3ʹ); IL-32γ (Forward primer, 5ʹ-AGGCCCGAATGGTAATGCT-3ʹ and reverse primer, 5ʹ-CCACAGTGTCCTCAGTGTCACA-3ʹ); TGF-β (Forward primer, 5ʹ-CTAATGGTGGAAACCCACAACG-3ʹ and reverse primer, 5ʹ-TATCGCCAGGAATTGTTGCTG-3ʹ). GAPDH ((Forward primer, 5ʹ-CTGACTTCAACAGCGACACC-3ʹ and reverse primer, 5ʹ-GTGGTCCAGGGGTCTTACTC-3ʹ) was used as a reference gene for normalization. The relative mRNA expression of study genes was calculated as 2^−△CT^ with △CT being the difference in threshold cycles for target and reference.

### Western blotting

Total protein was extracted from MM cells, and protein quantification was performed using the BCA method (Beyotime Institute of Biotechnology, Haimen, China). Protein samples were subjected to 10% SDS–polyacrylamide gel electrophoresis (SDS-PAGE). Proteins were then transferred onto 0.2 μm PVDF membranes and blocked with QuickBlock (Beyotime, China, P0252) for 30 min at room temperature. Next, the membranes were incubated overnight at 4 ℃ with the first antibody as indicated: visfatin (1:1000, ab236874, Abcam), BAX (1:1000, ab32503, Abcam), BCL-2 (1:1000, ab182858, Abcam), Caspase3 (1;1000, ab32351, Abcam), Caspase9 (1;1000, ab202068, Abcam), IκB (1;1000, T55026 F, Abmart), p-IκB (1;1000, TP56280 F, Abmart), NF-κB-p65 (1;1000, TP56372 F, Abmart), p-NF-κB-p65 (1;1000, TA2006 s, Abmart). After washing with TBST, the membranes were incubated with HRP-conjugated goat anti-rabbit IgG at room temperature for 1 h followed by washing with TBST [23]. The protein bands were visualized using chemiluminescence in a Tanon 5100 system(Tanon, China).

### In vivo xenograft tumor model

Female NOD-SCID mice (aged 6 weeks) were obtained from Jiangsu Collective Pharmachem Biotechnology Company (Jiangsu, China). The mice were randomly divided into two cohorts (n = 5) of sh-NC and sh-visfatin using the RPMI-8226 cell line. 4 × 10^6^ MM cells were dissolved in 150 μl PBS and injected into the skin of the right axilla of mice. Tumors and body weights were measured every three days. Tumor volume (mm^3^) was calculated by using the modified ellipsoid formula 1/2 (length × width × height). At the same time, body weight was measured, and when a tumor size increase of more than 2000 mm^3^ was observed, euthanasia was performed as a measure for reducing pain as early as possible. The central disruption (cervical dislocation) method was used as the method of euthanasia under anesthesia with isoflurane. After that, the tumor tissue was collected. All the procedures used in these experiments were approved by the Ethics Committee of Lanzhou University Second Hospital. All animal experiments were conducted in accordance with the Guidelines for endpoints in animal study proposals. The tumor size did not exceed the permitted maximum diameter of 20 mm and maximal volume of 2000 mm^3^[24].

### Immunohistochemical analysis

The xenograft tissues were fixed in 4% paraformaldehyde. Samples were stained with primary antibodies at 4 ℃ overnight. Secondary antibodies were incubated for 1 h. The stained samples were examined by microscopy, and representative sections were photographed. All results of the IHC staining score were calculated using Image J software and the IHC Profiler plugin. Ki-67 and IL6 expression were independently reviewed and scored by two clinical pathologists specializing in hematology. Different cases were re-examined until a consensus was reached. Ki-67 and IL6 expression were semi-quantitatively assessed using the IRS score, derived from the intensity score multiplied by the percentage of positive cells score. The intensity of immunostaining was scored on a scale of 0–3: no staining, 0; light yellow,1; brownish yellow, 2; and brownish brown, 3. The percentage of positive cells was scored on a 0–4 scale: less than 1%, 0; 1–10%, 1; 11–50%, 2; 50–80%, 3; 81–100%, 4 [25].

### Statistical analysis

Mapping and all statistical analyses were performed using GraphPad Prism 9.0 (GraphPad Software, Inc.). Data were presented as mean ± SD. For inter-group comparison, unpaired Student’s t-test or Wilcoxon rank-sum test was used dependent on whether data confirmed to a normal distribution. Similarly, Analysis of variance (ANOVA) or Mann–Whitney U test was performed for comparisons among multiple groups while correlation analyses were performed using Pearson correlation or Spearman correlation. To evaluate the diagnostic performance, receiver operating characteristic (ROC) analysis was performed. All statistical tests were two-tailed, and p-value < 0.05 was considered as significant (*P < 0.05, **P < 0.01, ***P < 0.001).

## Results

### Demographic and characteristics of MM patients and controls

The study included data from thirty-one MM patients and seventeen iron deficiency anemia patients excluding tumors. Demographic and clinical data including sex, BMI, and cytokines were given in Table 1. Among these indicators, IL-6 and IL-10 levels were significantly higher in MM patients compared to controls.

### Upregulation expression of visfatin in MM

**Fig.1:**
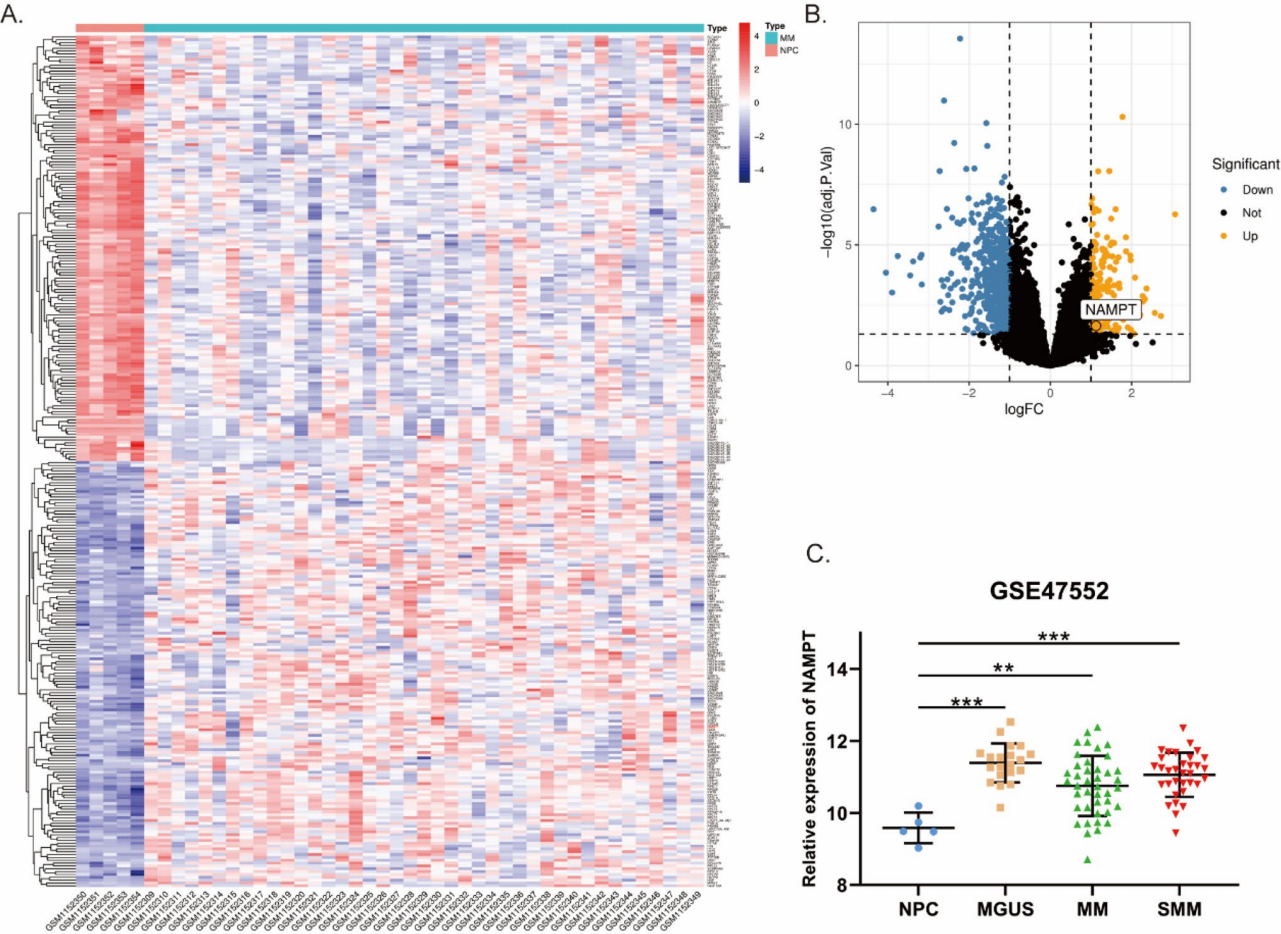
Nampt (the gene name of visfatin) is overexpressed in MM. **A** Heatmap representation of differential gene expression between MM and healthy controls. **B** Volcano Plot was used to visualize differentially expressed genes between MM and healthy controls. **C** Expression of Nampt in NPC, MGUS, SMM and MM. NPC: normal plamsa cells, MGUS: monoclonal gammopathy of undermined significance, SMM: smoldering multiple myeloma. *P < 0.05, **P < 0.01, ***P < 0.001

We obtained gene expression profiles of purified plasma cell samples from newly diagnosed patients with MM and healthy controls from GSE47552 dataset and analyzed DEGs using GEO2R. Nampt (the gene name of visfatin) expression was elevated in newly diagnosed patients. Nampt expression was also elevated in MGUS and SMM (Fig. 1). The differences were statistically significant (*P* < 0.05).

### The diagnostic value of visfatin in MM patients

**Fig.2:**
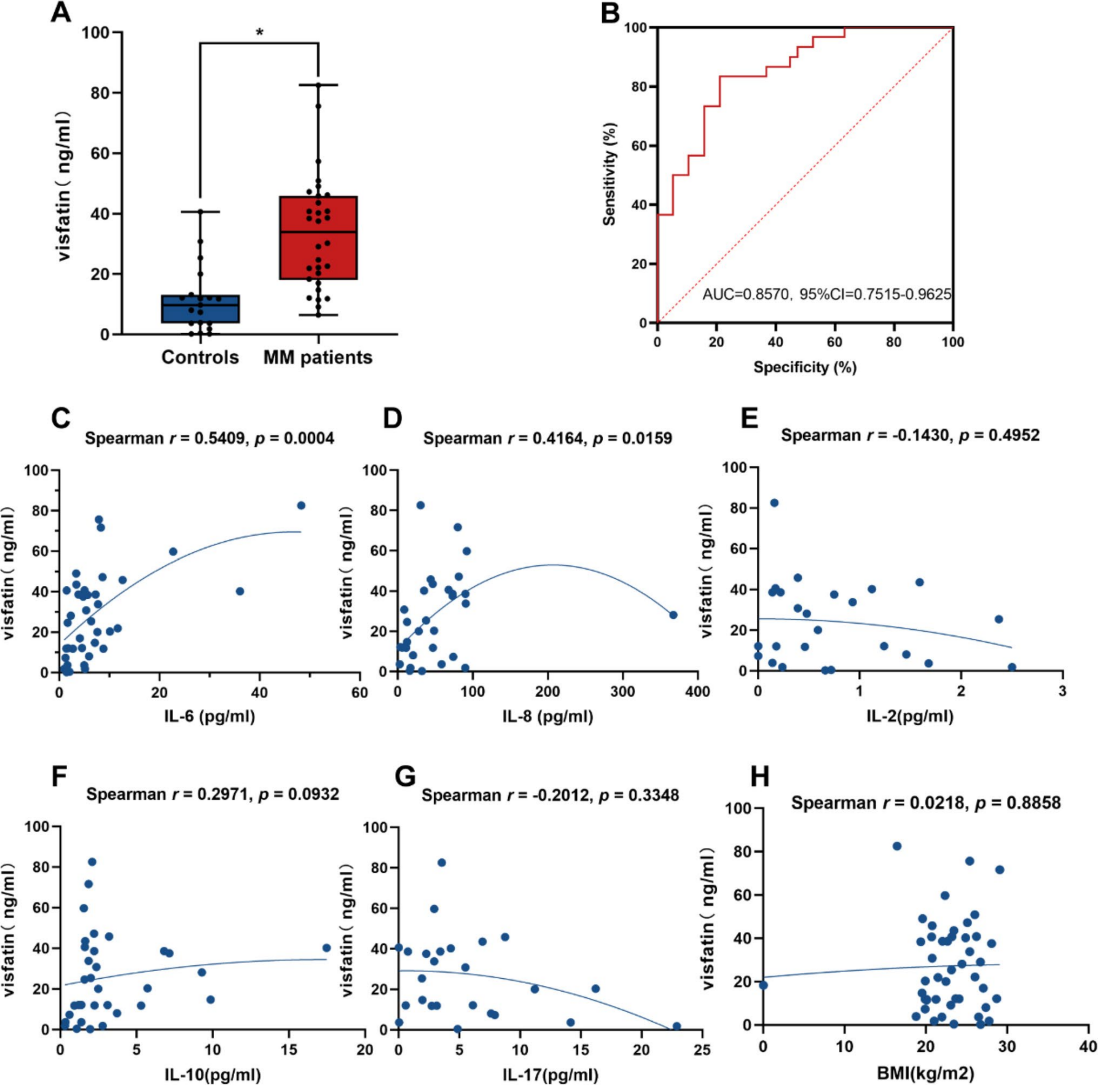
Diagnostic value of visfatin in MM patients. **A** Visfatin levels in bone marrow serum from MM patients and controls. Wilcoxon rank-sum test was used to analyze. **B** ROC analysis was performed to evaluate the performance of visfatin in distinguishing MM from controls. Correlation between visfatin and cytokines and BMI. **C**–**H** Correlation between visfatin and IL-6, IL-8, IL-2, IL-10, IL-17, and BMI. Correlation analyses were performed using Spearman correlation. *P < 0.05

To explore the role of visfatin in MM, visfatin in the bone marrow serum of MM was examined. As shown in Fig. 2A, the level of visfatin was significantly higher in the bone marrow of patients with MM in comparison with controls (*P* < 0.05), which is consistent with those of the database. ROC curve analysis was performed to evaluate the performance of visfatin in diagnosis between MM patients and controls. The area under the curve (AUC) was 0.8570, with a 95% confidence interval (CI) ranging from 0.7515 to 0.9625 (*P* < 0.05) (Fig. 2B). Furthermore, we analyzed the correlation between visfatin and cytokines. The results showed that visfatin was positively correlated with IL-6 (*P* = 0.0004) and IL-8 (*P* = 0.0159) (Fig. 2C, D). There was no correlation between visfatin and other cytokines we detected (Fig. 2E–H).

### Visfatin regulates the proliferation of MM cells

**Fig.3:**
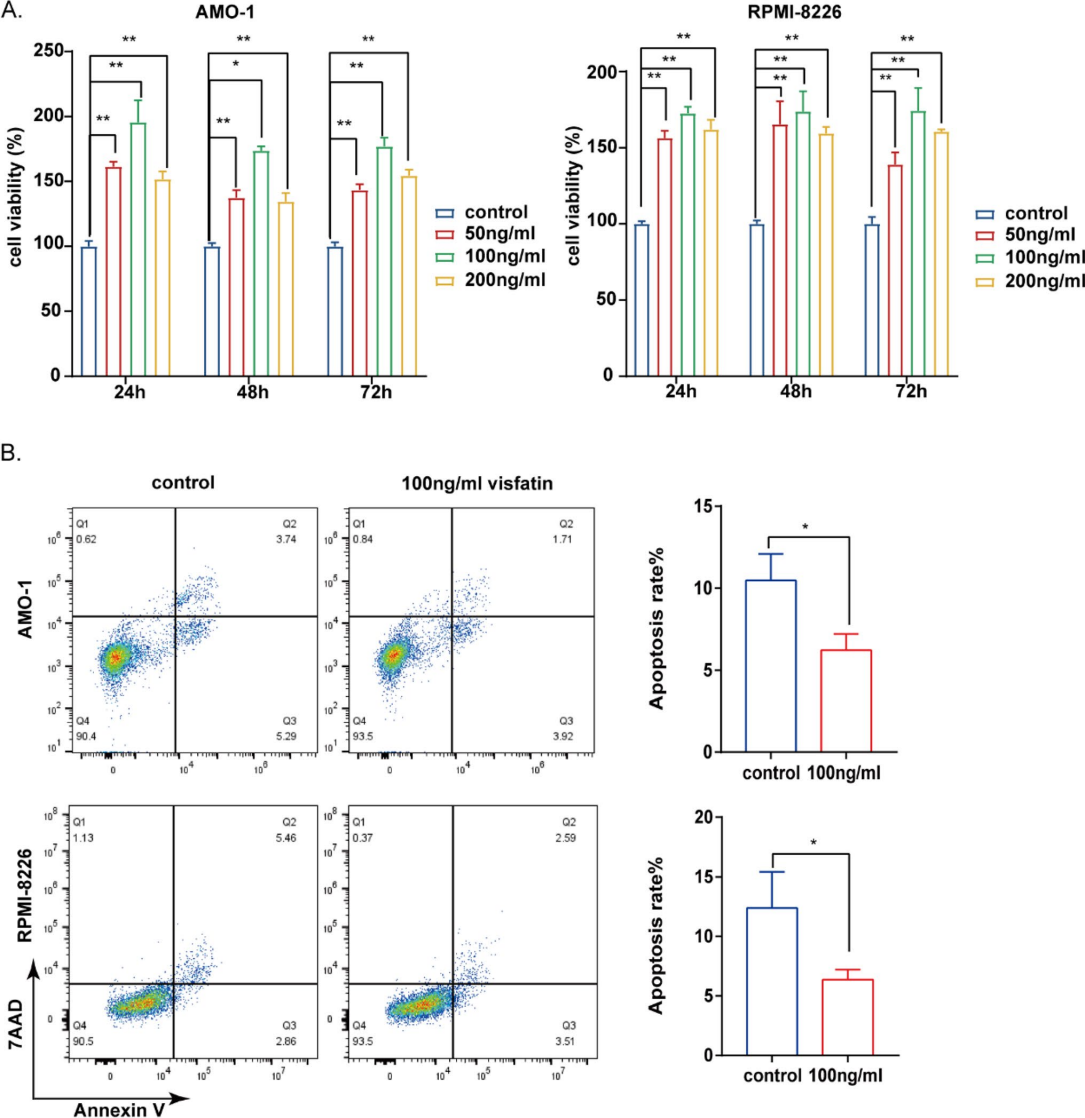
Visfatin regulates the proliferation and apoptosis of MM cells. **A** The CCK8 assay showed that visfatin regulated cell proliferation in AMO-1 and RPMI-8226 cell lines. Analysis of one-way ANOVA was performed for comparisons among multiple groups. **B** Flow cytometry to detect apoptosis rate in two cell lines treated with visfatin (100 ng/ml). Student’s t-test was used to analyze. *P < 0.05, **P < 0.01

RPMI-8226 and AMO-1 cells were treated with gradually increasing concentrations of visfatin for 24 h, 48 h, and 72 h. The CCK8 assay showed that visfatin promoted cell proliferation significantly in the 100 ng/ml group (Fig. 3A).The results showed a statistically significant difference (*P* < 0.05). Furthermore, to evaluate the survival capability of MM cells exposed to visfatin, we detected apoptosis by flow cytometry. As shown in Fig. 3B, we observed the apoptosis rate was reduced by visfatin (100 ng/ml) in two cell lines. The results showed a statistically significant difference (*P* < 0.05).

### Knocking down visfatin expression regulates the proliferation of MM cells

**Fig. 4:**
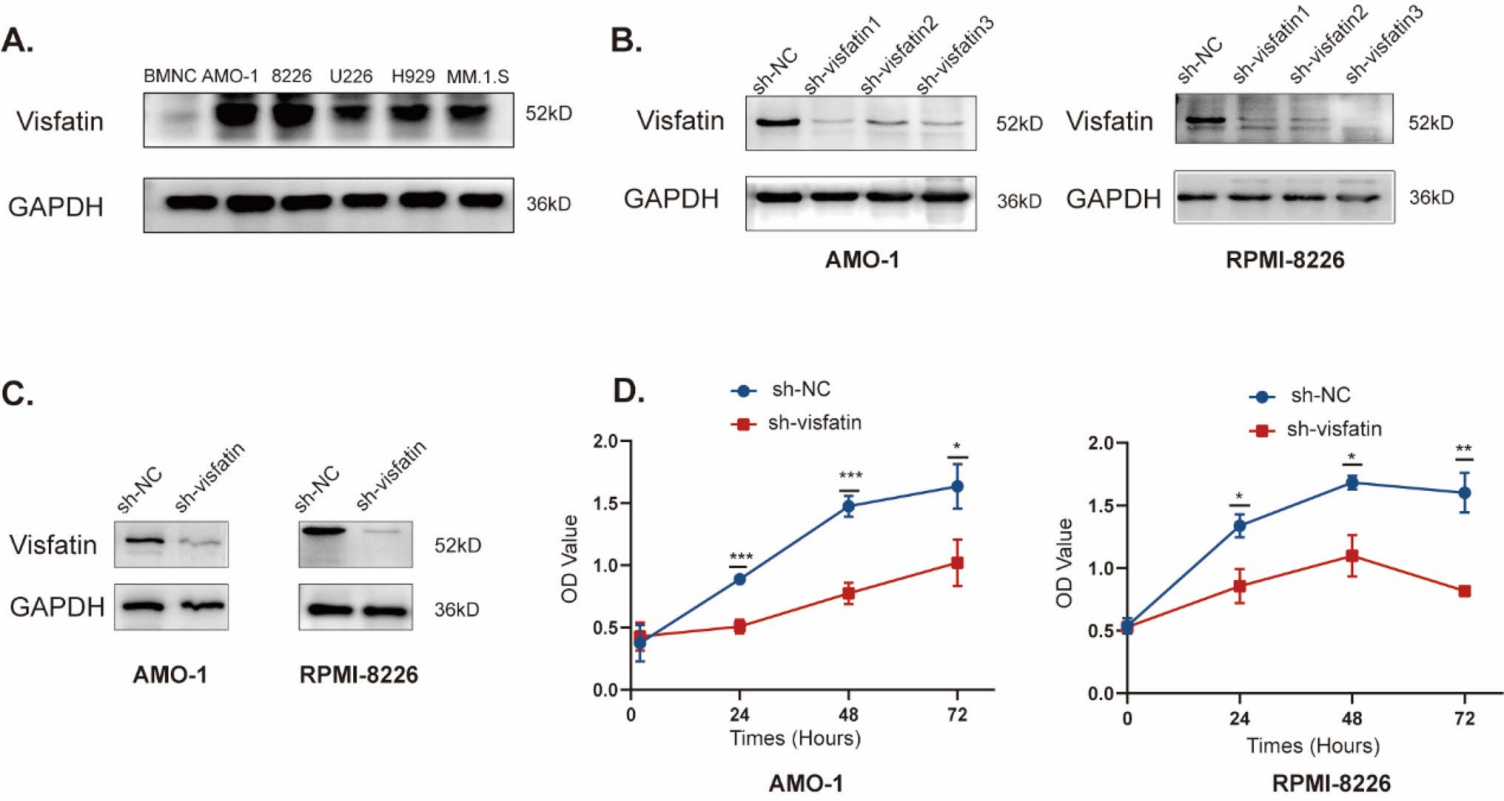
Knockdown of visfatin regulates the proliferation of MM cells. **A** Visfatin expression in different MM cell lines. **B** The knockdown efficiency of visfatin in AMO-1 and RPMI-8826 cells. **C** For the AMO cell line, sh-visfatin1was choosen, and for the RPMI-8226 cell line, sh-visfatin3 was choosen. **D** The OD values of the two cell lines were detected by CCK8. Student’s t-test was used to analyze. *P < 0.05, **P < 0.01, ***P < 0.001

Considering that visfatin was overexpressed in the bone marrow of MM patients, we asked whether visfatin be produced by MM cells. We examined the expression of visfatin in five cell lines (AMO-1, RPMI-8226, U266, H929, MM.1.S), and bone marrow mononuclear cells from controls using western blot. Visfatin expression was higher in MM cell lines (Fig. 4A). We choosed AMO-1 and RPMI-8226 to establish Visfatin-knockdown cell lines (sh-visfatin). After transfecting visfatin-targeting shRNA lentivirus in cells, we verified the expression of visfatin was decreased in sh-visfatin groups (Fig. 4B). We selected cells with the lowest visfatin expression, indicating the highest knockdown efficiency, for subsequent experiments. For the AMO cell line, we selected sh-visfatin1, and for the RPMI-8226 cell line, we chose sh-visfatin3. By using Western blot, we verified the knockdown efficiency of visfatin (Fig. 4C). In The OD value of sh-visfatin groups was lower than shNC groups in two cell lines by CCK8 assay (*P* < 0.05) (Fig. 4D).

### Knocking down visfatin expression regulates the apoptosis of MM cells

**Fig. 5:**
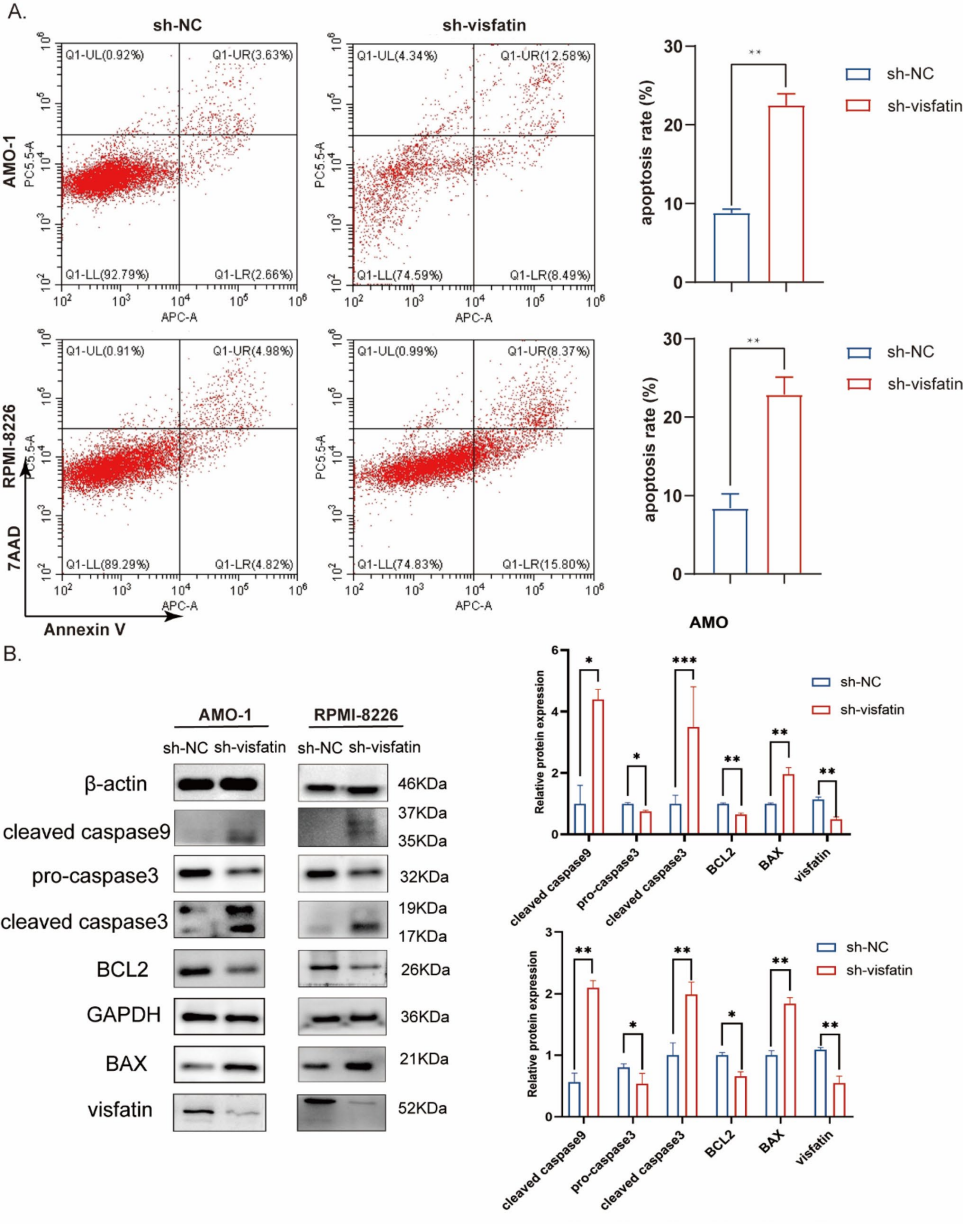
Knockdown of visfatin regulates the apoptosis of MM cells. **A** Flow cytometry to detect apoptosis rate in the sh-visfatin and sh-NC groups. Student’s t-test was used to analyze. **B** Western blot assay to detect the expression of BAX, BCL2, Caspase3, Caspase9 and visfatin. Student’s t-test was used to analyze.*P < 0.05, **P < 0.01, ***P < 0.001

We detected the apoptosis rate by flow cytometry. In two cell lines, the apoptosis rate of sh-visfatin groups were higher than shNC groups (Fig. 5A). The differences were statistically significant. In order to confirm the role of visfatin on apoptosis, anti-apoptotic (BCL2) and pro-apoptotic (BAX, cleaved caspase3, and cleaved caspase9) protein expression was analyzed by western blot. Western blot analysis showed the expression of BCL2 was significantly down-regulated in sh-visfatin groups compared to the shNC groups. Knockdown visfatin gene significantly up-regulated the expression of BAX, cleaved caspase3, and cleaved caspase9 (Fig. 5B). The results showed a statistically significant difference (*P* < 0.05).

### Knockdown visfatin regulates the expression of IL-6 in MM cells

**Fig. 6:**
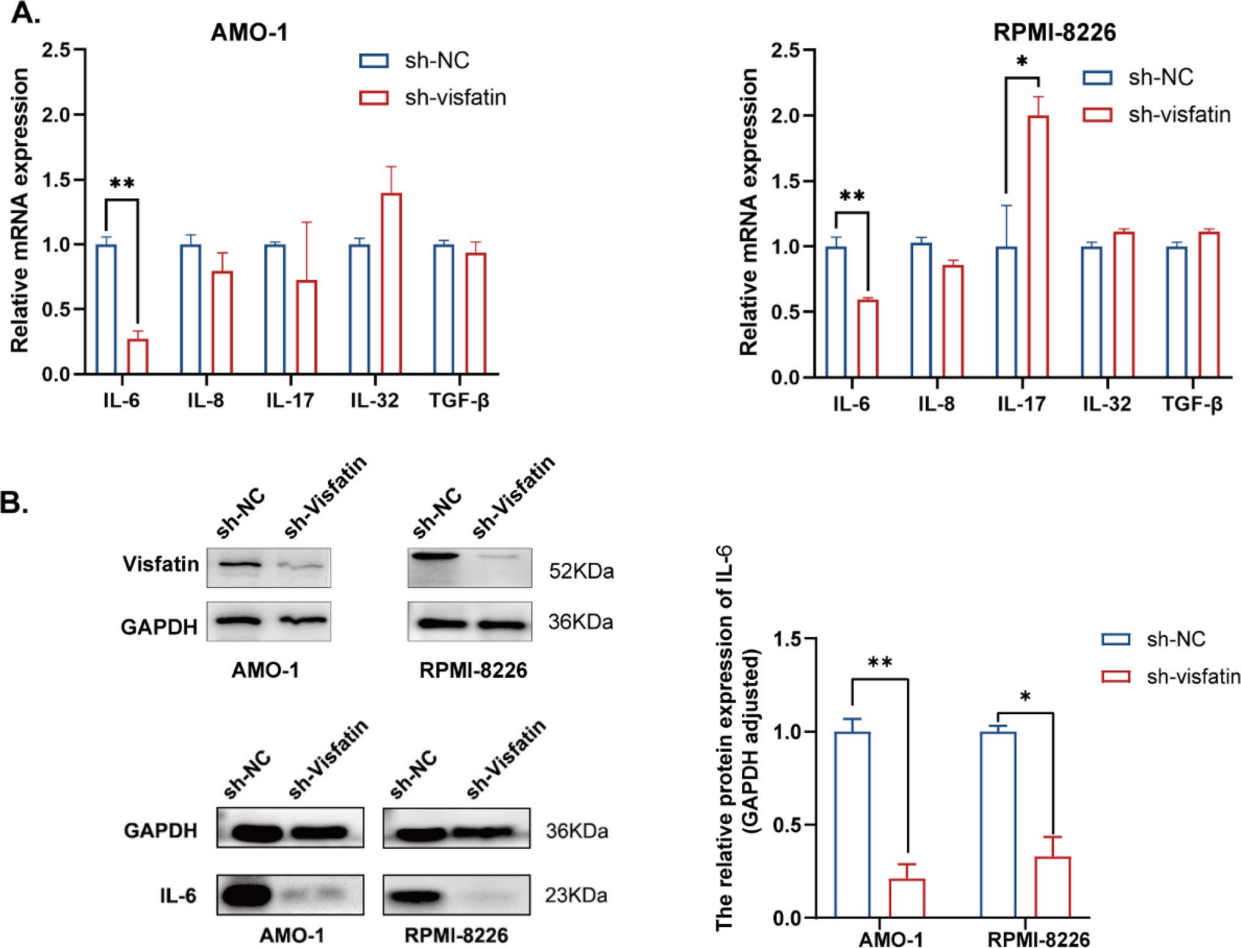
Visfatin regulates the expression of cytokines in MM cells. **A** The mRNA expression of IL-6, IL-8, IL-17, IL-32, and TGF-β in AMO-1 and RMI-8226 cell lines was tested by qRT-PCR. Student’s t-test was used to analyze. **B** The knockdown efficiency of visfatin and the expression of IL-6 in AMO-1 and RPMI-8826 cells were checked by western blot analysis. Student’s t-test was used to analyze. *P < 0.05, **P < 0.01

Several cytokines such as IL-6, IL-8, IL-17, IL-32, and TGF-β can regulate the malignancy of MM cells [26]. We tested the expression of these cytokines in AMO-1 and RPMI-8226 cells transfected with shNC or sh-visfatin. qRT-PCR showed that sh-visfatin can significantly decrease the expression of IL-6 in the two cell lines (*P* < 0.05). Knockdown of visfatin can increase the expression of IL-17 in RPMI-8226 cell line, but it had no significant effect in AMO-1 cell line. There is no significant effect on other measured cytokines (Fig. 6A). The sh-visfatin induced suppression of IL-6 in AMO-1 and RPMI-8226 was confirmed by western blot (Fig. 6B). The results showed a statistically significant difference (*P* < 0.05). These results showed that visfatin regulates the expression of IL-6 in MM cells.

### Visfatin can regulate IL-6 production via NF-κB signaling pathway

**Fig.7:**
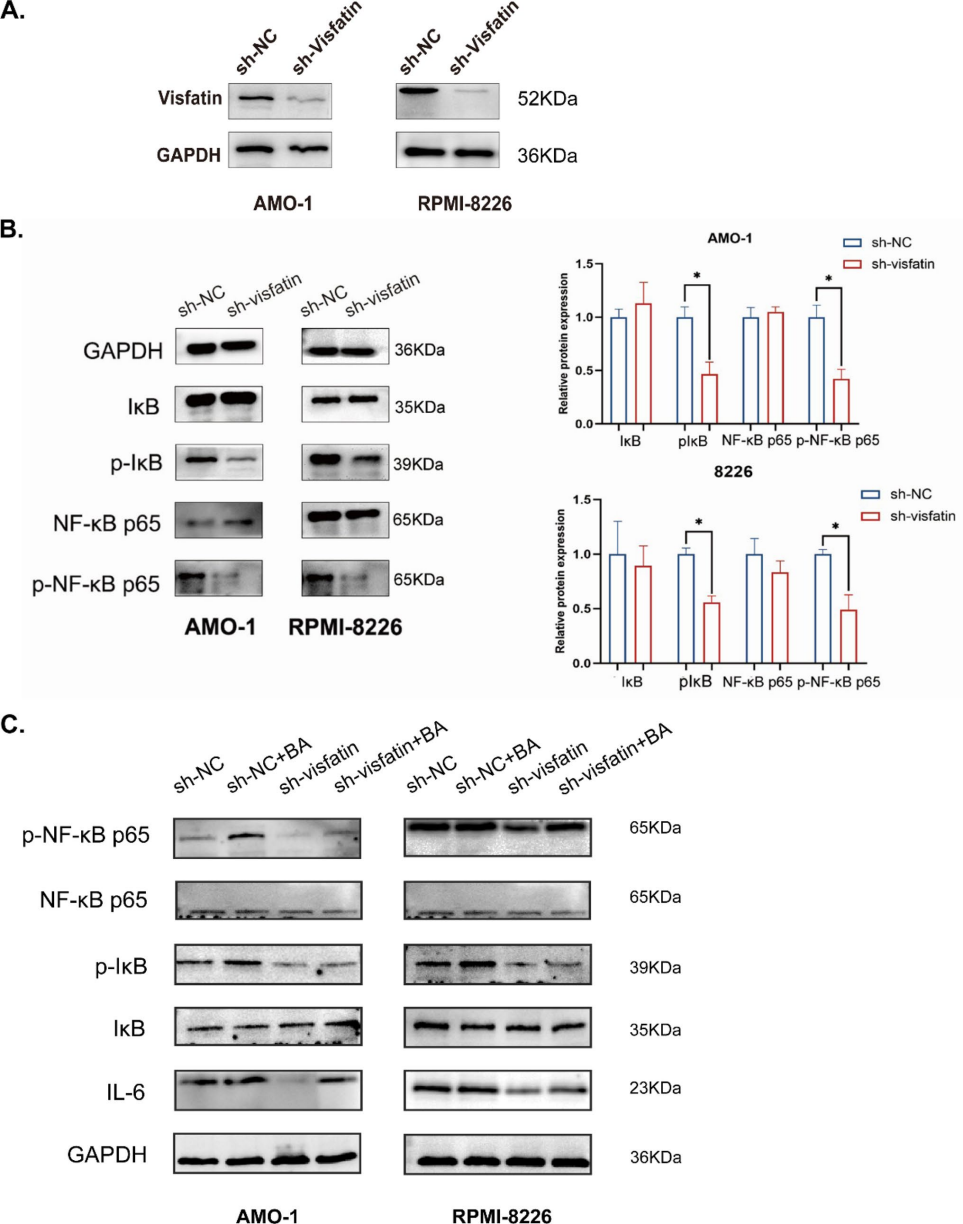
Signaling pathway involved in IL-6 production is regulated by visfatin in MM cells. Wilcoxon rank-sum test was used to analyze. **A** The knockdown efficiency of visfatin in AMO-1 and RPMI-8826 cells. **B** Phosphorylation IκB, IκB, phosphorylation NF-κB p65 subunit, and NF-κB p65 subunit were detected by western blot. **C** IL-6, phosphorylation IκB, IκB, phosphorylation NF-κB p65 subunit, and NF-κB p65 subunit in AMO-1 and RPMI-8226 cell lines treated with betulinic acid (BA) was measured by western blot. *P < 0.05

Several studies suggested visfatin played effects via the NF-κB signaling pathway [27–29]. To identify the signal pathway of the effect of visfatin on MM cells, we examined the expression of phosphorylation IκB, and phosphorylation NF-κB p65 subunit. Figure 7A showed the knockdown efficiency of visfatin by using Western blot. The results showed that knockdown visfatin led to a significant decreasement in the phosphorylation level of the IκB and NF-κB p65 subunit (Fig. 7B). We treated the cells with betulinic acid, a transcriptional activator of NF-κB pathway (ab120654, abcam, USA), to confirm the production of IL-6 via the NF-κB signaling pathway, and discovered that it counteracted the inbition of sh-visfatin group on IL-6 production (Fig. 7C). The results showed a statistically significant difference (*P* < 0.05).

### Knocking down visfatin expression attenuated MM cell proliferation in vivo

**Fig. 8:**
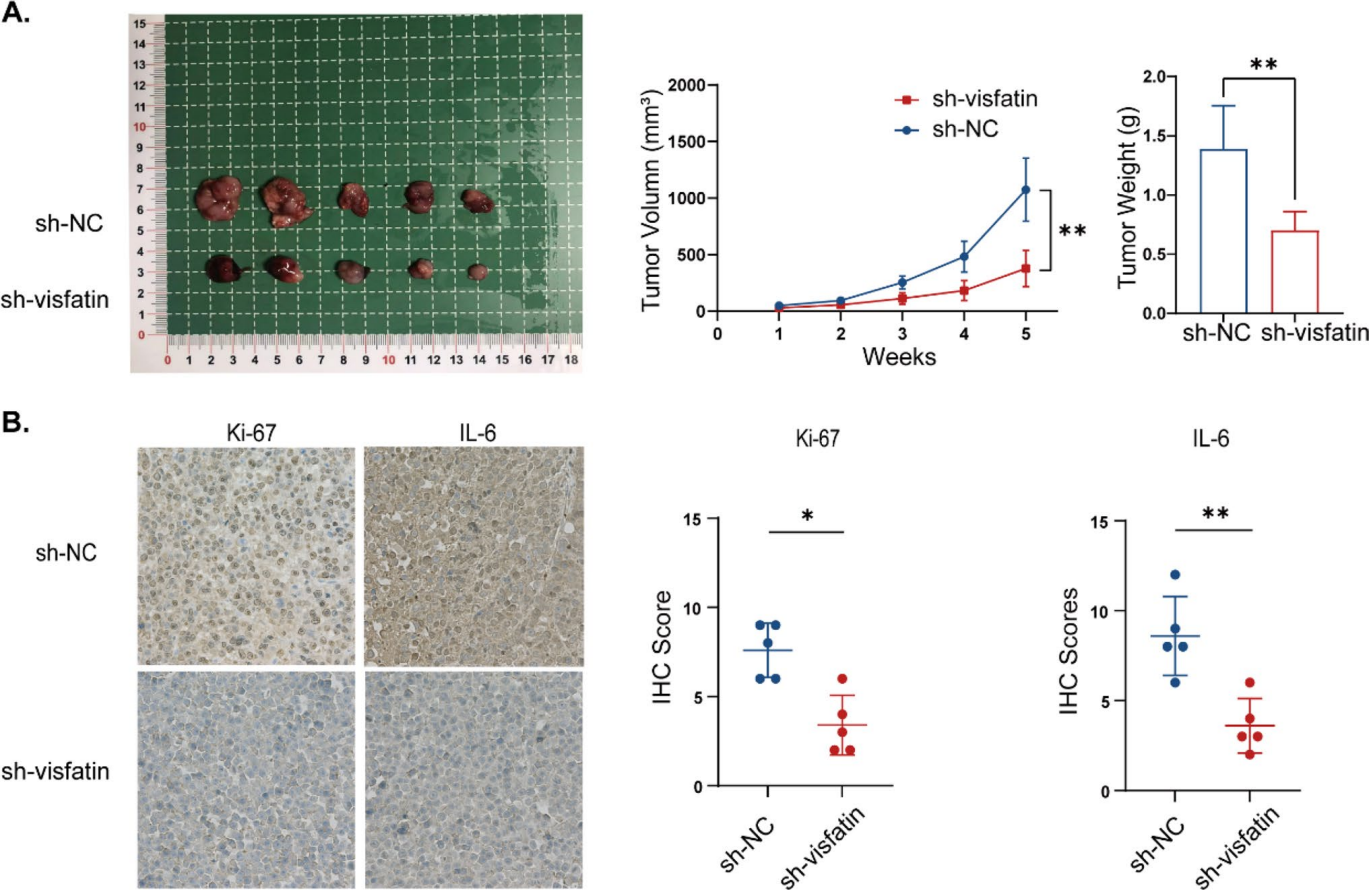
Visfatin promotes MM cell proliferation in vivo. Student’s t-test was used to analyze. **A** The size of xenograft tumors in each group of mice, the weight and volume of the transplanted tumors. **B** IHC staining of Ki67 and IL-6. *P < 0.05, **P < 0.01

Considering that visfatin was overexpressed in MM cells and the bone marrow serum of MM patients, we asked whether visfatin indeed plays roles in vivo. Ten female NOD-Prkdcem26 Cd52/Gpt mice were randomly divided into 2 groups to establish a xenograft tumor model. The tumor weight and volume in shNC groups were higher than those in sh-visfatin groups (Fig. 8A). We examined the effect of visfatin on Ki-67, and IL-6 by immunohistochemistry. The results showed that knockdown visfatin downregulated Ki-67, and IL-6 levels and with lower IHC scores (Fig. 8B). The results showed a statistically significant difference (*P* < 0.05).

## Discussion

The previous clinical studies suggested that visfatin is up-regulated in MM patients [30]. However, the role of visfatin in MM has not been fully elucidated. In this study, we first collected the clinical general data and test data of thirty-one patients with MM and seventeen controls. In accordance with the clinical diagnosis of multiple myeloma (MM), IL-6 and IL-8 levels were found to be significantly elevated in MM patients. Consequently, we collected serum from bone marrow to assess visfatin expression and investigate the protein's role in MM. The findings revealed that visfatin levels were higher than those in the control group. Additionally, we conducted ROC curve analysis to evaluate the diagnostic potential of visfatin in MM. The results indicate that visfatin may serve as a valuable diagnostic tool for MM.

Then, to further study the role of visfatin in MM, we demonstrated visfatin can be widely measured in MM cells. Visfatin has been associated with the progression of various cancer types [6], as supported by a growing body of evidence. Our current research suggests that visfatin may play a beneficial role in the proliferation of multiple myeloma (MM) cells, which is consistent with findings from studies on breast cancer, endometrial cancer, and renal cell carcinoma [31–33]. We observed that silencing visfatin expression in MM cell lines, specifically RPMI-8226 and AMO-1, impeded cell growth and heightened apoptosis. Thus, visfatin appears to promote cell growth while simultaneously inhibiting apoptosis. One of the primary growth factors driving myeloma development is IL-6. Myeloma cells produce IL-6 both autocrinely and paracrially, and this cytokine offers protection to MM cells [34]. Our findings indicate that visfatin may play a role in the development of MM by regulating IL-6 expression. Specifically, reducing visfatin levels could potentially decrease IL-6 expression. Additionally, we suggest that IL-6 might be responsible for the increased proliferation and prevention of apoptosis in MM cells induced by visfatin.

How does visfatin promote the production of IL-6 in MM cells? To verify this, we explored the molecular mechanism of IL-6 production induced by visfatin. We detected IκB and NF-κB p65 subunit and the phosphorylation of them. The results showed knockdown the expression of visfatin led to a dramatic decrease in the phosphorylation level of IκB and NF-κB p65 subunit. Several studies have experimented with betulinic acid as a transcriptional activator of the NF-kB pathway [35, 36]. We used known activator of NF-κB pathways to study the downstream signaling pathways of visfatin. The findings indicate that knocking down visfatin reduces IL-6 production, a process that may be reversed by activating the NF-κB pathway. Notably, earlier research and various studies conducted in different cell types suggest that visfatin can activate the NF-κB signaling pathway [37, 38]. Based on these results, we propose that visfatin utilizes the NF-κB signaling pathway to stimulate the production of IL-6 in multiple myeloma (MM) cells.

Our research confirms the critical role of visfatin through compelling in vivo xenograft experiments conducted in mice. By knocking down visfatin, we significantly reduced both the size and weight of subcutaneous tumors. Furthermore, immunohistochemistry demonstrated a notable decrease in the expression of IL-6 and Ki67, underscoring the importance of targeting visfatin in developing effective cancer therapies.

Prior research on visfatin has mainly targeted solid tumors, metabolic diseases, and their associated inflammation. Recently, attention has shifted towards the role of visfatin in hematological malignancies [32], revealing a significant gap worth exploring. Notably, multiple myeloma (MM) currently has no established diagnostic markers, and its pathophysiology remains largely elusive. Our study highlights the potential of visfatin as a novel diagnostic marker for MM, thereby offering a refreshing perspective on the disease’s underlying mechanisms.

**Fig. 9:**
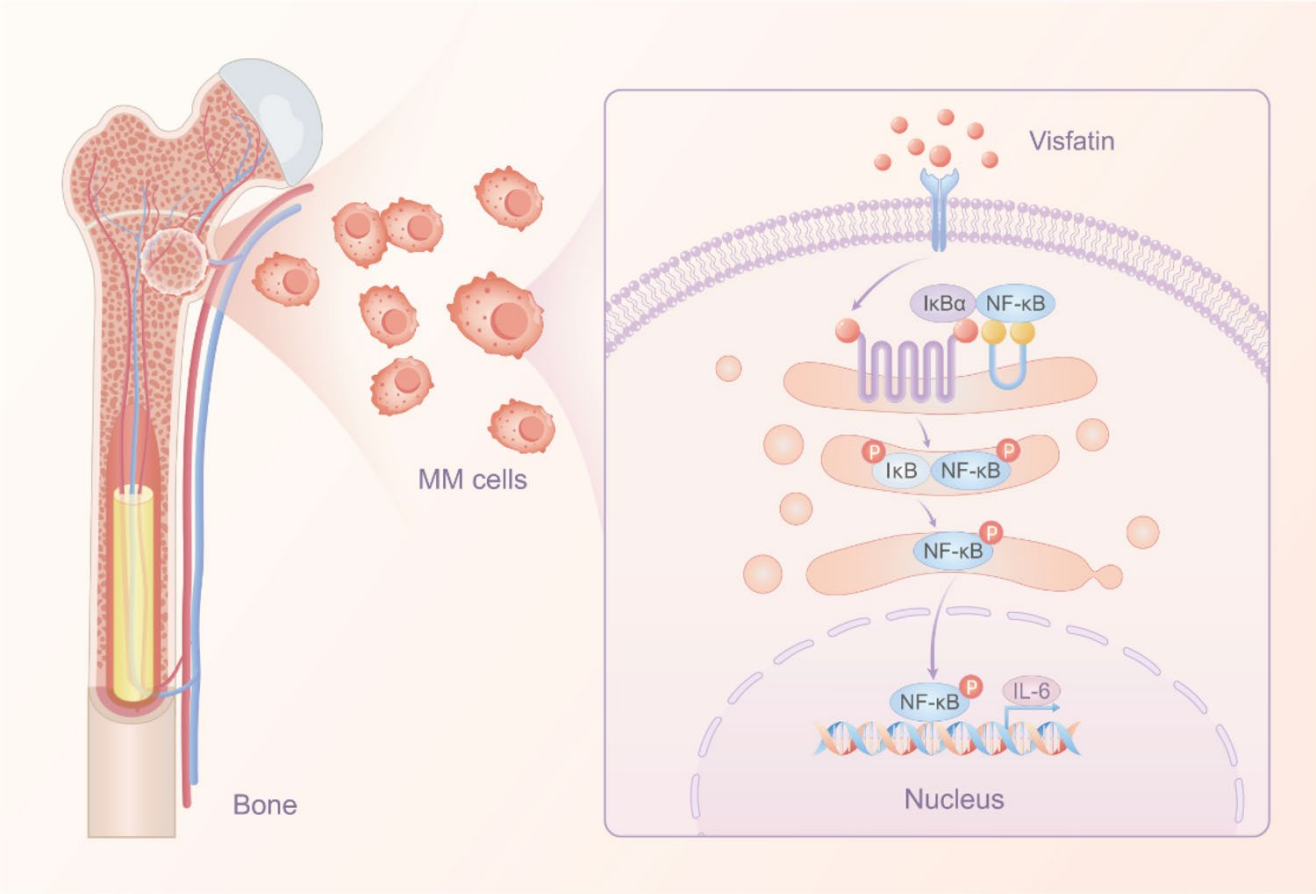
A schematic model for visfatin stimulated IL-6 production in MM cells

Importantly, further research is essential to unravel the specific role of visfatin in MM. Our findings suggest that visfatin may play a vital role in MM's pathophysiology by activating the NF-κB signaling pathway to stimulate IL-6 production (Fig. 9). This insight opens the door for innovative therapeutic approaches that target visfatin, paving the way for improved management strategies for multiple myeloma.

## Data Availability

The dataset supporting the conclusions of this article is included within this article and is available from the corresponding author upon request. No public data was used for the research described in this article, so we didn’t deposit any data associated with our research into a publicly available repository.
